# A Hybrid Distance Measure for Clustering Expressed Sequence Tags Originating from the Same Gene Family

**DOI:** 10.1371/journal.pone.0047216

**Published:** 2012-10-11

**Authors:** Keng-Hoong Ng, Chin-Kuan Ho, Somnuk Phon-Amnuaisuk

**Affiliations:** 1 Faculty of Computing and Informatics, Multimedia University, Cyberjaya, Malaysia; 2 Faculty of Creative Industries, Universiti Tunku Abdul Rahman, Petaling Jaya, Malaysia; Auburn University, United States of America

## Abstract

**Background:**

Clustering is a key step in the processing of Expressed Sequence Tags (ESTs). The primary goal of clustering is to put ESTs from the same transcript of a single gene into a unique cluster. Recent EST clustering algorithms mostly adopt the alignment-free distance measures, where they tend to yield acceptable clustering accuracies with reasonable computational time. Despite the fact that these clustering methods work satisfactorily on a majority of the EST datasets, they have a common weakness. They are prone to deliver unsatisfactory clustering results when dealing with ESTs from the genes derived from the same family. The root cause is the distance measures applied on them are not sensitive enough to separate these closely related genes.

**Methodology/Principal Findings:**

We propose a hybrid distance measure that combines the global and local features extracted from ESTs, with the aim to address the clustering problem faced by ESTs derived from the same gene family. The clustering process is implemented using the DBSCAN algorithm. We test the hybrid distance measure on the ten EST datasets, and the clustering results are compared with the two alignment-free EST clustering tools, i.e. *wcd* and *PEACE*. The clustering results indicate that the proposed hybrid distance measure performs relatively better (in terms of clustering accuracy) than both EST clustering tools.

**Conclusions/Significance:**

The clustering results provide support for the effectiveness of the proposed hybrid distance measure in solving the clustering problem for ESTs that originate from the same gene family. The improvement of clustering accuracies on the experimental datasets has supported the claim that the sensitivity of the hybrid distance measure is sufficient to solve the clustering problem.

## Introduction

Sequencing techniques have progressed rapidly in recent years, thus various types of sequence data have been produced and they are publicly available for research purpose. Despite many genome assemblies are available at present, research on expressed sequence tag (EST) is still on-going, due to it is a cost-effective resource for expression data analysis [1], [2], functional analysis [3], and single-nucleotide polymorphisms [4]. In addition, [5] claimed in their research work that conceptually translated ESTs can be used to predict subcellular location of protein. In general, ESTs are short single pass sequence reads derived from complimentary DNA (cDNA) libraries, and they can be produced in large quantities with inexpensive cost. Sanger-derived ESTs have typical lengths between 200–800 bases [6].

Since ESTs are short, they are unlikely to provide any useful gene information if they are unprocessed. One of the key steps in the EST processing pipeline is clustering. The objective of this step is to collect overlapping ESTs that originate from the same gene into a unique cluster. ESTs in the same cluster are then assembled to form a consensus sequence, which is important for gene identification. Several publicly available EST databases include Unigene [7], TIGR Gene Indices [8], STACK [9], and UCSC Genome Browser [10]. These databases have become the major sources of information for many academic research and laboratories.

For EST clustering algorithms, they can be broadly grouped into two classes, i.e. alignment-based clustering and alignment-free clustering. The first class relies on pair-wise alignment, and this alignment can either be transcript-based (EST-EST) or genome-based (EST-Genome). Transcript-based alignment compares similarities among ESTs, where BLAST [11] or its variant can be used to accomplish the task. PaCE [12], GMAP [13], ESTMapper [14], ECgene [15], and EasyCluster [16] are attached to genome-based alignment. These clustering tools generate better clustering quality as compared to the transcript-based, but these tools are only operational if the required genome assemblies exist. Therefore, it is not applicable for an organism whose genome has yet to be sequenced, especially for the new species.

In alignment-free clustering, *d2_cluster* [17] is an established algorithm for clustering ESTs and cDNAs. Pair-wise comparisons of ESTs are not dependent on alignment, but depend on the word occurrences between the sequences. This method implements a windowing strategy for subsequence comparisons between two ESTs. This strategy is based on the principle idea that two ESTs are clustered together if they have subsequences that are similar. It implies that ESTs that are overlapping with each other to a certain extent (subsequence length in this case), can be grouped into the same cluster. Hazelhurst et al. [18] introduced the *wcd* tool to cluster ESTs. It is a clustering method similar to *d2_cluster*. In fact, both methods apply the same distance measure and also implement the windowing strategy. The key differences between them include the efficient implementation of the basic quadratic algorithm, and the use of heuristics in *wcd* tool. Both of them are meant to speed up the computation time.

Recently, Rao et al. [19] proposed a tool called *PEACE*, to cluster ESTs. This tool also adopts the same distance measure and windowing like *wcd* and *d2_cluster*, but it uses the concept of minimum spanning tree to perform clustering. In terms of clustering quality, these alignment-free EST clustering methods are claimed to be competitive with each other. When they are compared with the alignment-based method, it is in fact a trade-off between speed and sensitivity. The alignment-free methods tend to deliver faster computation time with acceptable quality degradation. Another shortcoming of alignment-free methods is they are prone to produce unsatisfactory clustering results when they deal with ESTs originating from the same gene family. For instance, ESTs from different genes in the family might be clustered together by the alignment-free method and this will directly drag down the clustering accuracy.

In this paper, we have extended the EST clustering work using the alignment-free approach, with the aim to resolve the problem stated in the drawback. We propose an improved distance measure for EST clustering, where it has higher sensitivity than existing methods. The distance measure can be considered as a hybrid, since it is derived from the combination of local and global statistical metrics. Density-based clustering is selected for the clustering process. The experimental study involves ten datasets and they are evaluated with performance metrics. The results reveal that the proposed distance measure is capable to deliver competitive clustering accuracies for all tested datasets.

## Problem Statement

With the rapid growth in bioinformatics, there have been quite a number of published works focusing on the alignment-free distance measures for biological sequences. These distance measures can be generalized into several classes, and they are distance based on counting word frequencies [17], [18], [19], [20], [21], [22], distance based on compression [23], [24], and distance based on information theory [25], [26], [27]. Since EST is also classified as short biological sequence, therefore the above distance measures can be considered to be applied on ESTs. In fact, most of the alignment-free distance measures are actually extended from the word frequencies counting class.

In the previous section, we have briefly introduced several alignment-free EST clustering tools (*d2*_*cluster*, *wcd*, and *PEACE*). These highlighted clustering tools have the common distance measure named *d^2^*, and it originates from the word frequencies counting class. The key difference is the distance is measured within a fragment or subsequence of EST. The *d^2^* distance that incorporates the windowing approach has been claimed to perform reasonably well as compared to other alignment-free distance measures for ESTs. Hence this distance measure has been widely accepted in recent years. Furthermore, it has been embedded in the STACK tool [20], which is a platform to provide a complete analysis on express transcripts.

Even though the *d^2^* is a widely used distance measure in alignment-free EST clustering, it does have a drawback. In our research, we often use these tools to perform clustering on many EST datasets. Generally they perform quite well on majority of the datasets, but we found out that they tend to deliver unsatisfactory clustering results when they are dealing with ESTs that come from the same gene family. A Gene family is a set of homologous genes that are likely to have similar biochemical function [28]. According to [28]–[29], there are more than 10,000 gene families in the human genome, and the number of gene families that has been identified so far in the animals genomes has surpassed 14,000. Thus, it is very important to have a reliable method that can perform clustering accurately for ESTs that come from the same gene family. An example is the HOXA gene family that has 12 genes in the family. The possible reason behind the lower than expected clustering results might be due to the high similarities among the genes in the family, and the *d^2^* distance is not sensitive enough to differentiate them. In EST clustering, the aim is to assign EST to its original gene, but not to other gene (even though they come from the same gene family). Thus there is a need to enhance the current methods’ sensitivities in order to resolve the problem.

## Materials and Methods

**Figure 1:**
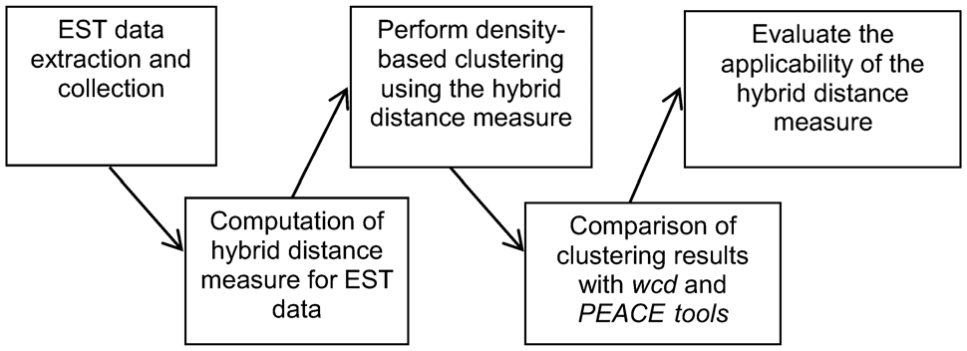
An overview of our experimental study on the proposed hybrid distance measure.

The section discusses our research method and experimental data, and an overview of the research work is shown in Figure 1. It begins with the extraction and collection of EST datasets using the UCSC genome browser, followed by the computation of our proposed distance measure for ESTs. The subsequent step involves clustering, which uses the generated EST distances as measure, and it is implemented using the density-based algorithm. The clustering results are then evaluated and analyzed thoroughly. The next step is the comparison of clustering results with the existing EST clustering tools, to evaluate the performance of our proposed distance measure. The last step involves the applicability study of the proposed distance measure on another clustering technique, which is hierarchical clustering. The following subsections describe the proposed hybrid distance measure, clustering algorithm, cluster validity index and experimental datasets in details.

### The Proposed Hybrid Distance Measure, hbd_EST

In the previous section, we have highlighted several EST clustering tools that are implemented using the alignment-free approach. The aspects discussed cover not only their distance measures and clustering methods, but also their advantages and drawbacks. With the intention to resolve the problem encountered in the clustering tools that implement the window-based *d^2^* distance, we propose an improved distance measure (*hbd_EST*) to cluster ESTs. This alignment-free distance measure combines two features that are locally and globally extracted from ESTs. In this case, the global features refer to the patterns or characteristics that can be found in the entire sequence of EST. On the contrary, patterns or characteristics that appear only in a portion/subsequence of EST are considered as local features. The principle idea is that the decision for grouping two ESTs into a single cluster must not be solely based on the similarity comparison between subsequences, but have to consider global feature as well.

**Figure 2:**
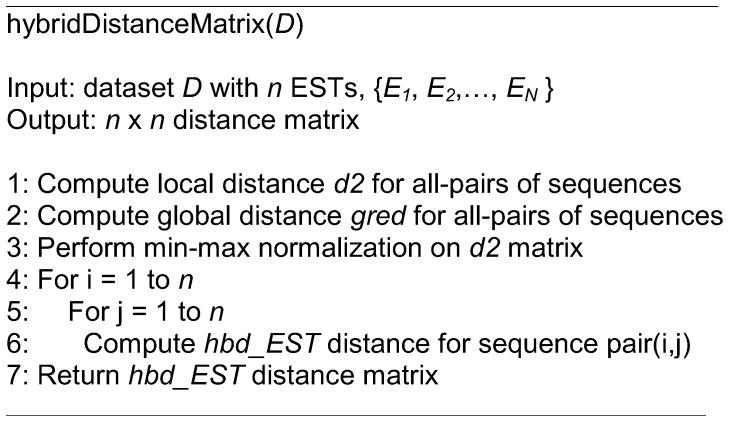
The algorithm for computing the *hbd_EST* distance measure.

To the best of our knowledge, there has yet to be any published work that introduces an alignment-free distance measure, where it is measured by combining the local and global features of EST. With the *hbd_EST* distance measure, we aim to improve the clustering accuracy for datasets that contain ESTs from the same gene family. At the same time, we would also like to find out to what extent, both features affect the clustering quality. The first thing to highlight is the local feature of EST. It can be derived using statistical methods on any portions/fragments of EST. Word counting in a defined window size is selected as the local feature of *hbd_EST* distance measure. The basic idea is that two similar sequences will have common words to a certain extent, or we can say that there are some common patterns that appear in sequences that are similar.

The similarity comparison based on word counting can be quantified using the well-known distance measure called *d^2^*. It was first proposed by [22], and later it is widely accepted and embedded into the established EST clustering tools such as *d2_cluster* [17], *wcd* [18] and *PEACE* [19]. We choose the same *d^2^* as the distance metric for our local feature, due to its reliability and acceptability. The use of this distance measure is also in-line with our main goal, which is to enhance the current methods’ sensitivity, so that the problem faced in the gene family dataset can be resolved.
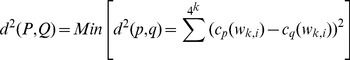
(1)


Equation (1) shows how the *d^2^* distance between two ESTs, *P* and *Q* is computed. Let *d^2^*(*p, q*) denotes the distance between *p* and *q*, where *p* is a subsequence from *P* and *q* is a subsequence from *Q*. *w_k,i_* is a word with length *k*, and the maximum number of distinct words (with length k) is 4*^k^*. The number of occurrences of *w_k,i_* in *p* and *q* are represented by *c_p_* (*w_k,i_*) and *c_q_* (*w_k,i_*). Since one EST can be partitioned into many subsequences, therefore a lot of *d^2^* distances can be produced. As such, it will only pick the smallest *d^2^* value as the distance for *P* and *Q*. For word length and window size, we use the default values (word length = 6, window size = 100 bases) set in both clustering tools (*wcd* and *PEACE*). It is because we need to make a fair and unbiased comparison of clustering accuracy between our proposed distance measure (*hbd_EST*) and both of them.

As we refer to the alignment-free distance measures available for biological sequences, we are aware that a majority of them measure the distances based on the whole sequence length. Thus we believe that the feature that is extracted globally plays an inevitable role in sequence comparison, and cannot be omitted even in short sequences like ESTs. This motivated us to propose an idea to combine the local and global features in our *hbd_EST* distance measure. The global feature in our case is derived from the measurement of sequence relative entropy, which is an important concept in both statistical biology and information theory. Earlier research works [27], [30], [31] proposed the distance measures based on the relative entropy, and the authors claimed that their results were comparable to the alignment-based similarity distance measure.

**Figure 3:**
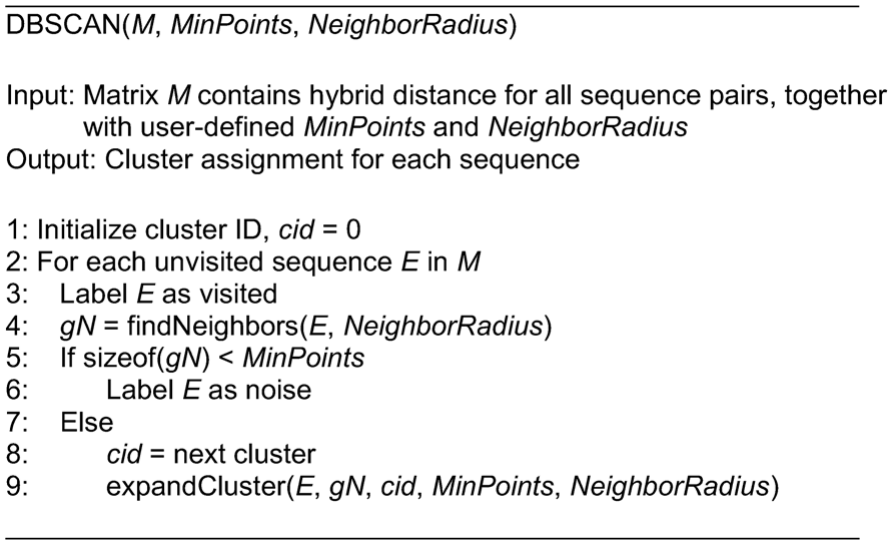
The pseudo codes for the *DBSCAN* function.

We explore the global feature of ESTs by adopting the relative entropy approach, and the similarity between ESTs is quantified using the distance called generalized relative entropy, *gred* [25]. It is a statistical measure based on the frequencies of words with length *k*. The *gred* distance between two ESTs, *P* and *Q* can be obtained using the formula shown in Equation (2). In the equation, *w_k_*_,*i*_ denotes the word *i* with size *k*, and the maximum possible words is 4*^k^*. The frequencies of word *i* in sequence *P* and *Q* are denoted in *f^ P^* (*w_k,i_*) and *f^ Q^* (*w_k,i_*). The use of word frequency is considered to be more appropriate than the word count since ESTs may have different lengths. Therefore, the impact of length bias can be minimized. This range of the *gred* distance is between 0 and 1. The distance is 0 when two identical ESTs are compared, and 1 for two completely different ESTs.
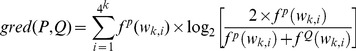
(2)


Both distances that quantify the local and global features of ESTs are to be combined to form a hybrid distance. Prior to this step, we have to ensure that both distance values fall into the [0,1] range and data normalization will be performed when it is required. We therefore perform min-max normalization on the *d^2^* distance since its original value is out of this range. Min-max normalization is a linear transformation that preserves the relationships among the original data values [32]. Equation (3) shows the transformation of *d^2^* distance using the min-max normalization. In the equation, *d^2^* (*P*, *Q*) is the original distance for the EST pair, *P* and *Q*. The maximum and minimum values of the original data are denoted with *d^2^_max_* and *d^2^_min_*, while the new maximum and minimum are represented by *d^2^_new_max_* and *d^2^_new_min_*. In this case, it is 1 for the new maximum and 0 for the new minimum.
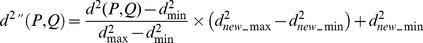
(3)


Once both values are in the same range, the hybrid distance (*hbd_EST*) can then be computed. In this work, we conduct an in-depth analysis to study the influence of the local and global features towards the clustering accuracy, especially on the dataset that contains ESTs from the same gene family. As such, the *hbd_EST* distance is calculated by combining both distances with different weight, with the condition that the sum of weights is 1. For instance, the *hbd_EST* distance can be generated from the combination of 0.95 local (*d^2^*″) and 0.05 global (*gred*). The formula to calculate the *hbd_EST* is shown in Equation (4). Let *d^2^″*(*P*, *Q*) and *gred*(*P*, *Q*) represent the normalized *d^2^* distance and the *gred* distance for the EST pair, *P* and *Q*. *A* and *B* are the weights with possible values in {0, 0.05, 0.1,…, 0.9, 0.95, 1}. The value of *hbd_EST* is between 0 and 1, where 0 indicates two identical ESTs and 1 means two ESTs have 0% similarity.
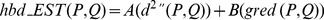
(4)


Figure 2 shows the algorithm for computing the *hbd_EST* distance. The algorithm takes dataset containing *n* ESTs as input, and the expected output is the hybrid distance matrix with *n* × *n* size. The steps involved in this algorithm can be summarized in the following: Step 1 is the computation of local distance for all EST pairs, followed by generating global distance in step 2. The subsequent step involves the normalization of local distance, while the last step is the computation of *hbd_EST* distance by combining both local and global distances at user-defined weight. The hybrid distance matrix is then used for clustering, where the density-based is the selected clustering algorithm.

**Figure 4:**
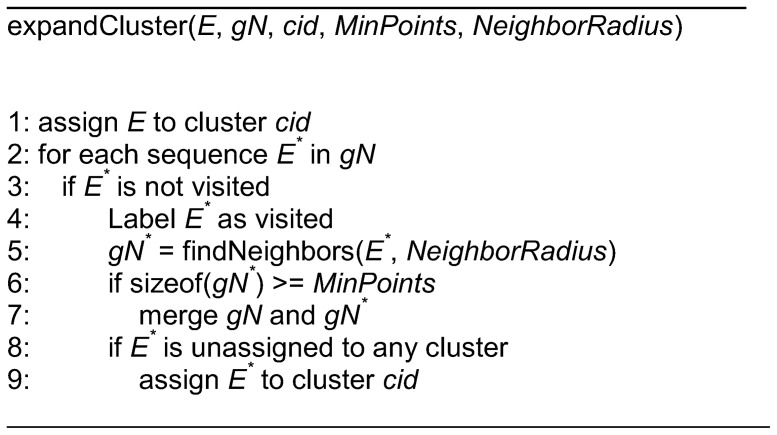
The pseudo codes for the *expandCluster* function.

### Density-based Clustering

Density-based clustering is a partitioning clustering algorithm, and its general idea is the formation of clusters is based on the density of objects in the data space. A region with a high density of objects is regarded as a cluster, and the clusters are separated by regions of low object density. Several established density-based clustering algorithms include OPTICS [33], CLIQUE [34], and DBSCAN [35]. These clustering algorithms have been demonstrated to deliver consistent and accurate results, therefore the work has been extended by many researchers and quite a number of variants [36], [37], [38], [39] have been proposed.

In our work, the clustering of ESTs is performed using the DBSCAN. This clustering algorithm is chosen due to two reasons. Firstly, the cluster can be in arbitrary shape or size. Secondly, the number of clusters is not required to be determined prior to clustering. The two factors are justifiable in EST clustering. This is because information such as cluster shape/size and cluster quantity is unavailable for any EST dataset before the clustering takes place. The basic idea of DBSCAN is that each point in a cluster must contain at least a set of points within a given radius (both can be user-specific). It implies that the neighborhood density has to exceed a user-specified threshold. Generally, the DBSCAN algorithm can be explained with two functions, i.e., *DBSCAN* and *expandCluster*.

In the *DBSCAN* function, it takes three parameters, i.e. distance matrix, minimum neighbors, and neighborhood radius. The cluster id (*cid*) is initially set to 0, and then it picks an unvisited EST (*E*) and marks it as visited. The next step is to find the EST’s neighbors (*gN*) within the user-specified radius. The EST is labeled as a singleton (noise) if the number of neighbors is less than the minimum neighbors (*MinPoints*). On the contrary, it forms a cluster using a next cluster id, and proceeds to expand the cluster (*expandCluster* function) when the number of neighbors exceeds the minimum neighbors. The clustering process terminates when all ESTs are visited. The details for the *DBSCAN* function are shown in Figure 3.

**Figure 5:**
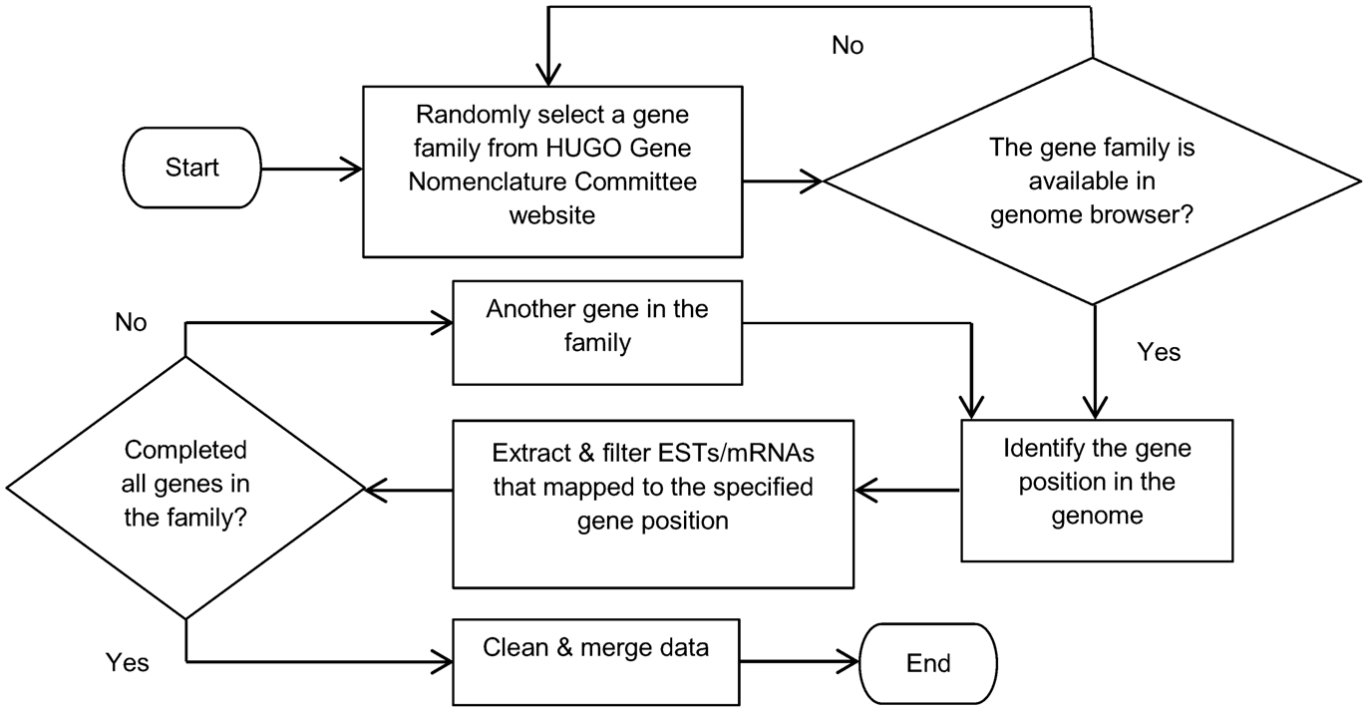
The flow chart diagram for the extraction of EST datasets using the Genome Browser.

In the *expandCluster* function, the expansion begins with the neighbors (*gN*) of the EST found in the *DBSCAN* function. An unvisited neighbor (E*) is then picked and marked as visited. It is followed by searching the EST’s neighbors within the neighborhood radius. The neighbors (gN*) of this EST will be merged with *gN* if the quantity is greater or equal to the minimum neighbors (*MinPoints*). This EST will be assigned with the current cluster id (*cid*) if it is still unattached to any cluster. The above steps are repeated until all ESTs in the *gN* have been visited. This clustering algorithm assigns each EST with a cluster membership, and it gives -1 to EST that does not belong to any cluster (singleton). Figure 4 shows the pseudo codes for the *expandCluster* function.

### Cluster Validity Index

In order to evaluate the performance of our proposed distance measure, the validity of the clustering results obtained from the experiments are assessed. We have chosen the Jaccard index [40] as the cluster validity index. It is a statistical method based on external criteria. It measures how good the clustering algorithm is, by matching cluster labels with externally supplied class labels. This validity index can also be used to measure the similarity of the clustering results associated to two different methods. In EST clustering, this index is quite commonly used and has appeared in several papers [18], [19], [41]. The authors in these papers used it to make comparisons between their proposed algorithms with other EST clustering tools.

**Table 1:** The details of the ten datasets extracted using the tools in the genome browser.

Dataset with	Total	Total ESTs/	Largest	Smallest
Gene Family	Clusters	mRNAs	Cluster Size	Cluster Size
Name	(Genes)		(ESTs)	(ESTs)
CYP2	16	1848	256	3
APOBEC	10	846	226	34
T-box	13	842	320	7
WNT	18	1410	395	4
CYP4	12	970	203	7
HOXA	12	711	203	3
PLXN	9	2712	741	128
CHRN	16	1014	168	3
SPDY	10	529	229	8
EPH	14	2202	479	28

Equation (5) shows how the Jaccard index can be measured from the clustering result. Consider {*C_1_*, *C_2_*… *C_m_*} is the clustering result of method *C* on a dataset, and *P_d_* is a defined partition of the dataset. In our case, variable *a* refers to the number of pairs of ESTs that belong to the same cluster in *C* and also to the same group of partition *P_d_*. While variable *b* denotes the number of pairs of ESTs that belong to the same cluster of *C*, but to a different group of *P_d_*. Variable *c* is the number of pairs of ESTs that belong to a different cluster of *C*, but to the same group of *P_d_*. The index has a value range of 0–1, which means that the index is 1 if both clustering methods produce an identical clustering structure on a dataset.
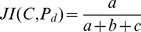
(5)


### Experimental Data

To evaluate the effectiveness of the proposed distance measure, ten EST datasets were extracted from the UCSC genome browser [10]. In brief, this genome browser is developed and maintained by the Genome Bioinformatics group, and it is attached to the University of California Santa Cruz. The reasons for selecting this website for downloading our datasets are its available easy-to-use tools, reliable data source, and complete annotations for various types of sequences. Furthermore, the mapping of ESTs/mRNAs to the specific gene can be visualized in a diagram. This feature enables a user to verify the correctness of the downloaded data easily.

**Table 2:** Six combinations with different weights between the local and global features.

Combination	Local	Global
No.	Feature	Feature
	Weight	Weight
CB1	0.95	0.05
CB2	0.90	0.10
CB3	0.85	0.15
CB4	0.80	0.20
CB5	0.75	0.25
CB6	0.70	0.30

Figure 5 shows the process flow of the data extraction. First, we refer to the website maintained by the HUGO Gene Nomenclature Committee [42] and browse through its gene family page. We randomly select a gene family from the published list, followed by checking the existence of the gene family in the genome browser. The process continues if the gene family is available, with the identification of a gene position in the human genome. On the other hand, we have to randomly choose another gene family again if it is not found in the genome browser. Once the gene location is known, we proceed to extract ESTs and mRNAs that are mapped to this gene location using the table browser [43]. The table browser is one of the tools found in the UCSC genome browser, and we utilize some controls available in this tool to filter and extract the data that we require.

Filtering is vital since we need to collect high quality and reliable ESTs and mRNAs. In our case, we download ESTs/mRNAs that are not only mapped to the specified gene location, but also intersecting with at least 80% of the RefSeq [44] genes. RefSeq (Reference Sequence) is provided by the National Center for Biotechnology Information (NCBI). It serves as a stable gene reference for annotation, identification, mutation, polymorphism analysis and expression studies.

**Figure 6:**
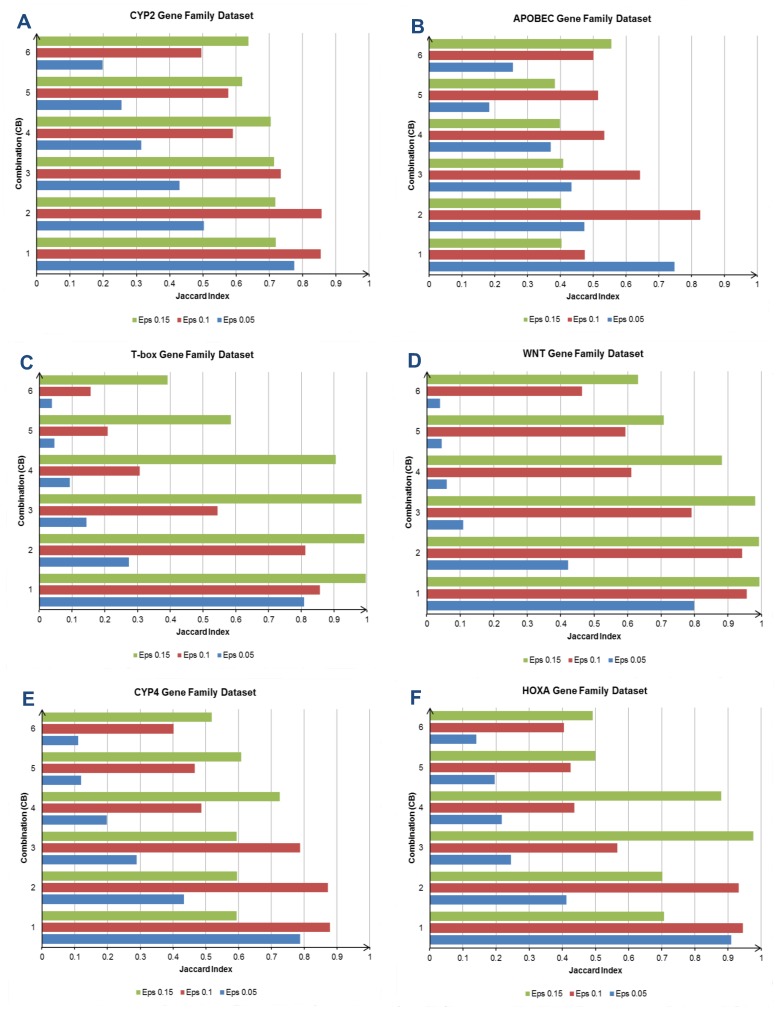
The clustering results (Jaccard Index) for the six experimental EST datasets. Graph (A) represents the clustering result for the CYP2 dataset. The best performer is the test with combination CB2 and neighborhood distance (Eps) 0.1. (B) is the clustering result for the APOBEC dataset, and the highest clustering accuracy is also achieved by the test with CB2 and Eps 0.1. The clustering result for the T-box dataset is plotted in (C). The test with CB1 and Eps 0.15 scores the highest clustering accuracy in this dataset. (D)–(F) show the clustering results for the WNT, CYP4 and HOXA datasets. Three tests (CB1–CB3, all with Eps 0.15) yield clustering accuracy above 0.98 in the WNT dataset. In the CYP4 dataset, the test with CB1 and Eps 0.1 records the highest clustering accuracy. The test with CB3 and Eps 0.15 outperforms other tests in the HOXA dataset.

We also mask the repeats found in the ESTs/mRNAs. This is to avoid the repetitive elements found in the data, from affecting the clustering outcome. For another gene in the family, we repeat the same steps, and it terminates when all the genes data are completely downloaded. In the final stage, we clean the data by removing any ESTs/mRNAs with a length shorter than 100 bp, and then form the dataset by merging the data from all genes. To ensure the correctness of the data, we verify all ESTs/mRNAs with their mapping locations using the genome browser. In our work, we have extracted ten datasets for experimental study, and their details are displayed in Table 1.

**Figure 7:**
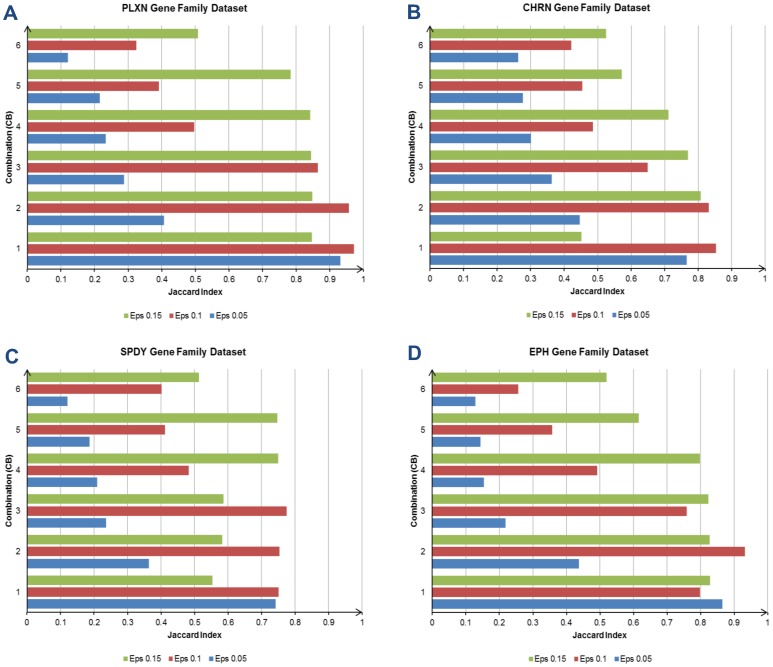
The clustering results (Jaccard Index) for the other four experimental EST datasets. (A) shows the plotted clustering result for the PLXN. The test with combination CB1 and neighborhood distance (Eps) 0.1 is the best performer in this dataset. The second highest clustering accuracy is recorded by the test with CB2 and Eps 0.1. The clustering result for the CHRN dataset is displayed in (B). The test with CB1 and Eps 0.1 sets the highest clustering accuracy (0.8527) in this dataset, while the test with CB2 and Eps 0.1 delivers the second best clustering accuracy. (C)–(D) denotes the clustering results for the SPDY and EPH datasets. In SPDY dataset, the highest clustering accuracy is achieved by the test with CB3 and Eps 0.1. It is followed by the test with CB2 and Eps 0.1. For the EPH dataset, the best clustering accuracy is done by the test with CB2 and Eps 0.1. The test with CB1 and Eps 0.05 produces the second best clustering accuracy.

## Results

In order to evaluate the effectiveness of our proposed *hbd_EST* distance measure in solving the problem highlighted in problem statement section, ten EST datasets have been used in the experimental study. Each dataset contains ESTs derived from the same gene family. We study the performance of the *hbd_EST* distance measure with different combinations of the following parameter values: Local feature weight, global feature weight and neighborhood distance in DBSCAN. These key parameters play crucial roles in this context. This is because an optimized weight between the local and global features, or/and a good neighborhood distance may improve the EST clustering outcome significantly.

**Table 3:** The parameters values used by the *hbd_EST* in comparative study.

Word	Window	Global	Local	Eps in
Size	Length	Feature	Feature	DBSCAN
	(bases)	Weight	Weight	
6	100	0.1	0.9	0.1

The correctness of our clustering result is evaluated based on the EST libraries from the genome browser, where the libraries are constructed based on the alignment of ESTs on the human genome assembly [45]. This means that we are comparing our clustering result with the alignment-based method, thus using other alignment-based clustering method for comparison is not essential in this case. On the other hand, the same datasets are tested with two alignment-free EST clustering tools (*wcd* & *PEACE*) and their clustering results are then compared and discussed in this section.

### Influence of Key Parameters on Clustering Results

The study on the weight covers the range of 0.05–0.30 for the global feature, and 0.70–0.95 for the local feature. The weight for the local feature is always greater than the global feature in all combinations. This is because the local feature plays a more significant role in EST clustering [46], [47], and the global feature is incorporated to enhance its sensitivity for solving the gene family problem. Table 2 describes the weight between both features in detail, and there are six combinations (CB1–CB6) with various weights used in the experiments. For the neighborhood distance used in the DBSCAN, we have tested the range from 0.05 to 0.3. The experimental results of the EST datasets with various settings are plotted in Figure 6 and 7 with ten graphs. Each graph represents the clustering results obtained from a single EST dataset. We will first observe and comment on the clustering results for each dataset, and then we will summarize and discuss the findings based on the overall clustering results from all the experimental datasets.

**Table 4:** The comparative evaluation of our proposed hybrid distance measure (*hbd_EST*), with the *wcd* and *PEACE* clustering tools.

Dataset	Jaccard Index/Number of Generated Clusters		
	*hbd_EST*	*wcd*	*PEACE*
CYP2	**0.8577**	0.7166	0.6908
(16 clusters)	15	11	10
APOBEC	**0.8256**	0.4030	0.4030
(10 clusters)	10	6	6
T-box	0.8125	**0.9895**	0.5235
(13 clusters)	18	13	13
WNT	0.9426	**0.9869**	0.5604
(18 clusters)	20	18	11
CYP4	**0.8721**	0.5927	0.5059
(12 clusters)	15	7	5
HOXA	**0.9329**	0.7133	0.5343
(12 clusters)	14	10	8
PLXN	**0.9568**	0.8451	0.3558
(9 clusters)	14	8	5
CHRN	**0.8314**	0.7295	0.4516
(16 clusters)	20	15	14
SPDY	**0.7538**	0.5752	0.5538
(10 clusters)	7	6	5
EPH	**0.9214**	0.8257	0.8262
(14 clusters)	19	16	16

The clustering result for the first dataset (CYP2 gene family) is displayed in Figure 6A. Based on the graph, the highest clustering accuracy (in Jaccard index) is 0.8577, and the second best delivers a marginally lower accuracy than the highest, which is 0.8546. Combination CB2 (0.1-global, 0.9-local) with the neighborhood distance (Eps) 0.1 is the setting that yields the best accuracy in this dataset. Meanwhile, combination CB1 (0.05–global, 0.95–local) with the same Eps is the setting that produces the second highest accuracy. For APOBEC gene family dataset (Figure 6B), the best accuracy (0.8256) is recorded by the combination CB2 with Eps of 0.1. The second best accuracy comes from the combination CB1 with Eps of 0.05, and its accuracy (0.7483) is moderately lower than the best one. In the T-box dataset (Figure 6C), there are three settings that deliver clustering accuracy above 0.98. They are the combination CB1–CB3, and all with Eps of 0.15. The best accuracy (0.9972) is obtained by the combination CB1, and subsequently the second best accuracy (0.9933) is achieved by the combination CB2. The combination CB3 gives 0.9842 for clustering accuracy, which is marginally above 0.98.

The WNT gene family (Figure 6D) is the fourth experimental dataset, and we observed that there are also three tests with accuracies exceeding 0.98. The parameter settings in these three tests are in fact identical with the best three clustering accuracies obtained by the tests on the T-box dataset. The three settings (combination CB1–CB3, all with Eps of 0.15) yield 0.9935, 0.9931 and 0.981 accuracy respectively. For the CYP4 dataset (Figure 6E), the best clustering accuracy (0.8793) is obtained by the combination CB1, with Eps of 0.1. It is followed by the combination CB2, with Eps of 0.1. This combination is able to score 0.8721 for the accuracy, which is slightly lower than the best clustering result in this dataset. The clustering result for the HOXA dataset is plotted in Figure 6F, and the best accuracy (0.9764) belongs to the combination CB3, with Eps of 0.15. The combination CB1, with Eps of 0.1 delivers the second best accuracy (0.9448) in this dataset.

The clustering results for the PLXN and CHRN datasets are plotted in Figure 7A and 7B.The *hbd_EST* performs the best with combination CB1 and Eps of 0.1 in the PLXN dataset. The recorded clustering accuracy is 0.9721. The second best clustering accuracy (0.9568) is set by combination CB2 with Eps of 0.1. In the CHRN dataset, the highest clustering accuracy is 0.8527 and it is scored by the test with combination CB1 and Eps of 0.1. The test with combination CB2 and Eps of 0.1 also shows encouraging result. It yields 0.8314 for the clustering accuracy, which is marginally lower than the best performer. Figure 7C and 7D display the clustering results for the remaining two experimental datasets, i.e. SPDY and EPH. The highest clustering accuracy (0.7756) in the SPDY dataset is obtained by the test with combination CB3 and Eps of 0.1. It is followed by the test with combination CB2 and Eps of 0.1, where it achieves 0.7538 for the clustering accuracy. In the EPH dataset, the best two clustering accuracies are 0.9314 and 0.8654 respectively. The highest clustering accuracy is recorded by the test with combination CB2 and Eps of 0.1, while the test with combination CB1 and Eps of 0.05 produces the second best clustering accuracy.

We can generalize some outcomes or findings based on the clustering results from all these datasets. Firstly, all datasets seems to perform satisfactorily when the weight is set at the range of 0.05–0.15 for the global feature, and 0.85–0.95 for the local feature. The clustering performance degrades when we increase the weight for the global feature. As a result, the experiment is not extended to higher weight (0.3 and above) for the global feature. This finding explicitly indicates that the local feature plays a more significant role than the global feature. Nevertheless the role of the global feature cannot be neglected even though its contribution is not substantial. This is because we have conducted tests using only the local feature, but none of them shows comparable clustering result with the combination of local and global features at the specified weight.

Another finding is the neighborhood distance used in the DBSCAN. We discovered that the optimized neighborhood distance is 0.1±0.05 for all datasets. It is because the best clustering result for each dataset is recorded within this range. The clustering results with higher neighborhood distances (0.2, 0.25 or 0.3) generally underperform the clustering results of the optimized range. We analyze this scenario and found that ESTs from different genes are merged together into a single cluster if the neighborhood distance is set to 0.2 or higher. The optimized neighborhood range (0.1±0.05) is an ideal range to be used in the DBSCAN for solving this clustering problem.

**Table 5:** The p-values of the t-test.

	p-value (t-test)	
	*wcd*	*PEACE*
*hbd_EST*	0.0314	0.000055

Both p-values (0.0314 and 0.000055) are below the alpha of 0.05. These p-values indicate significant differences between the clustering results of *hbd_EST*, with *wcd* and *PEACE.*

**Table 6:** The hybrid distance measure (with the same parameters in Table 3) is tested with another clustering algorithm, the hierarchical clustering.

Dataset	Jaccard Index	
	DBSCAN	Hierarchical
	Clustering	Clustering
CYP2	0.8577	0.8590
APOBEC	0.8256	0.8256
T-box	0.8125	0.9010
WNT	0.9426	0.9426
CYP4	0.8721	0.8652
HOXA	0.9329	0.9262
PLXN	0.9568	0.9622
CHRN	0.8314	0.8427
SPDY	0.7538	0.7448
EPH	0.9214	0.9229

Almost all experimental EST datasets (except T-box dataset) show similar clustering accuracies between the DBSCAN and the hierarchical clustering methods.

### Comparison of Clustering Results with Alignment-free EST Clustering Tools (wcd and PEACE)

We use the two widely accepted alignment-free EST clustering tools, i.e. *wcd* and *PEACE* to perform clustering on the same experimental datasets. Both of them are run using the default parameters set in the clustering tools (word size = 6, window length = 100 bases). These default parameters are used because they are the optimized and recommended values in both tools [19], [46]. To ensure the comparison is done in a fair and unbiased manner, we use the same word size and window length for the *hbd_EST* distance measure. Furthermore, we also use the same values for the global-local weight and the neighborhood distance (Eps) across all the ten datasets. Based on the results presented in the previous subsection, the following parameters values (Table 3) will be used for this comparative study.

Whenever we compare *hbd_EST* to *wcd* or *PEACE*, we are actually making comparison to the *d^2^* distance measure used by *wcd* and *PEACE*. Table 4 displays the comparison results for the ten EST datasets. In terms of clustering accuracy, the performance of *hbd_EST* is relatively better than *wcd*, with higher clustering accuracies achieved in eight out of ten experimental datasets. However, the clustering quality delivered by the *hbd_EST* in WNT dataset is slightly lower than *wcd*, and the clustering accuracy achieved by *wcd* is higher in the T-box dataset. When the comparison is made between the *hbd_EST* and *PEACE*, it indicates that the former yields higher clustering accuracies than the latter in all experimental datasets. From the comparison table, it can be observed that *wcd* also performs better than *PEACE* in most of the datasets (except the APOBEC and EPH datasets).

In the CYP2 dataset, our clustering accuracy is 0.8577 and it is higher than w*cd* (0.7166) and *PEACE* (0.6908). Besides that, the number of clusters generated by the *hbd_EST* is 15, and it is very close to the reference library (16 clusters). For both EST clustering tools, they generate 11 and 10 clusters respectively. For the APOBEC dataset, the *hbd_EST* has improved the accuracy rate substantially. It manages to score 0.8256, as compared to 0.4030 recorded in both clustering tools. In fact, the number of clusters formed by the *hbd_EST* is also the same with the reference library. The *hbd_EST* also scores higher clustering accuracies than the two clustering tools in the CYP4 and HOXA datasets.

**Figure 8:**
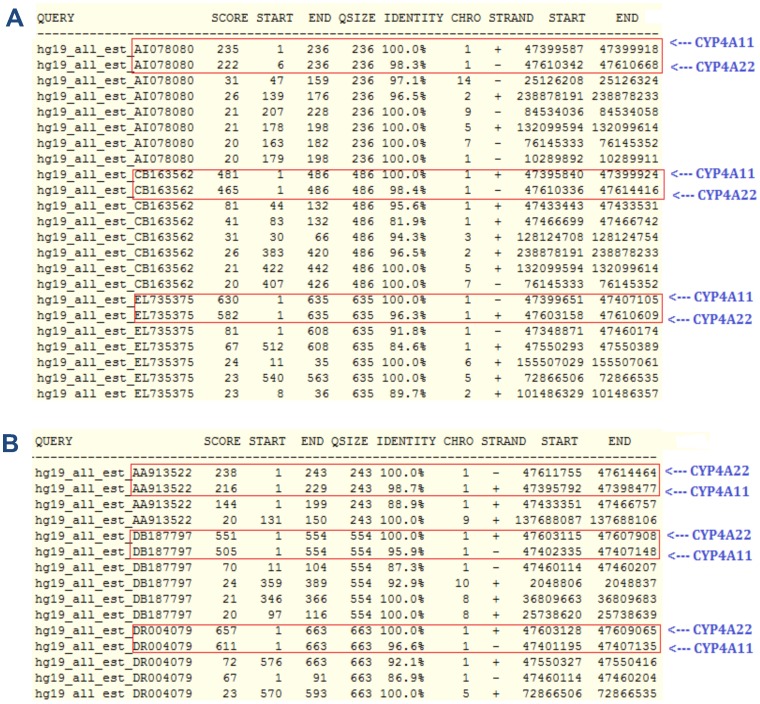
The BLAT scores for (A) three ESTs from CYP4A11 gene and (B) three ESTs from CYP4A22 gene. (A) indicates all three ESTs (AI078080, CB163562, EL735375) obtain highest BLAT scores (235, 481, 630) in their own gene, and record their second highest BLAT scores in the CYP4A22 gene. (B) shows the BLAT scores obtained by the three ESTs (AA913522, DB187797, DR004079) from the CYP4A22 gene. These three ESTs also yield the highest BLAT scores in their own gene, and their second highest BLAT scores can be traced in the CYP4A11 gene.

In the CYP4 dataset, the clustering accuracy recorded by the *hbd_EST* is 0.8721. This clustering result is very encouraging when compares to 0.5927 in *wcd* and 0.5059 in *PEACE*. The formation of clusters is 15 in the *hbd_EST*, and it is higher than the reference library (12 clusters). This situation happens when two larger clusters are divided into two or three smaller clusters. In this dataset, both EST clustering tools produce less than expected number of clusters. The preliminary finding has indicated that some ESTs from different genes are grouped together to form larger clusters. For the HOXA dataset, the clustering accuracy achieved by the *hbd_EST* is 0.9329 with formation of 14 clusters, and it performs better than the *wcd* and *PEACE*. The recorded clustering accuracies for both clustering tools are 0.7133 and 0.5343 respectively.

For the WNT dataset, the clustering accuracy obtained by the *hbd_EST* is satisfactory (0.9426) but slightly lower than *wcd*, where the latter scores the highest clustering accuracy (0.9869) among the three methods. The number of clusters generated by *wcd* is also identical to the reference library (18 clusters). The formation of clusters is 20 in the *hbd_EST*, and the investigation has found out that the extra clusters are actually caused by the division of clusters into smaller clusters. In the T-box dataset, *wcd* outperforms other two methods with clustering accuracy of 0.9895. At the same time, it also generates the same number of clusters with the reference library (13 clusters). The clustering accuracy (0.8125) obtained by the *hbd_EST* in this dataset is considered satisfactory, even though it is lower than *wcd*.

The performance (in terms of clustering accuracy) of the *hbd_EST* is quite consistent for the remaining four experimental datasets. The comparison of clustering results between the *hbd_EST*, *wcd* and *PEACE* on these four datasets has indicated that the *hbd_EST* performs better than the two EST clustering tools. Based on the clustering results, the *hbd_EST* manages to yield the clustering accuracy above 0.90 for the PLXN and EPH datasets. In the PLXN dataset, the recorded clustering accuracy by the *hbd_EST* is 0.9568. It scores the clustering accuracy of 0.9214 in the EPH dataset. The clustering results generated by the *wcd* on these two datasets are 0.8451 and 0.8257 respectively. For the CHRN dataset, the highest clustering accuracy (0.8314) is obtained by the *hbd_EST*, and both EST clustering tools yield 0.7295 and 0.4516 for the clustering accuracies. In the SPDY dataset, the clustering accuracy generated by the *hbd_EST* is 0.7538, and similar clustering accuracies are observed in *wcd* (0.5752) and *PEACE* (0.5538).

Generally, it is observed that the number of clusters generated by the *hbd_EST* distance measure is higher than the number of clusters found in the original data. Therefore, we analyzed each clustering result for the *hbd_EST* in detail. Investigation into the memberships of these extra clusters reveals that the higher than expected number of clusters is caused by the subdivision of a cluster into two or three smaller clusters. These smaller clusters contain only its own ESTs and they do not merge with ESTs from other clusters. Thus, they only have minimal effect on the clustering accuracy. In clustering, a subdivision of a cluster into several smaller clusters could happen when the clustering criteria become more stringent [48], [49]. The *hbd_EST* is considered more stringent in this case, since it compares the global and local features of EST sequences (*wcd* and *PEACE* only compare the local feature). As a result, the *hbd_EST* tends to subdivide a cluster into smaller clusters if it manages to detect the difference between ESTs in a gene.

We are aware that the performance of the *hbd_EST* is worse than the *wcd* in the T-box dataset. In view of this, we are interested to find out the possible reasons. Thus, we conduct an investigation into this matter. The main focus of the investigation is to study and analyze sequence (or subsequence) similarity among ESTs. In our case, we perform multiple sequence alignments on the ESTs in the T-box dataset using the *ClustalW* [50] tool. This is an analysis tool that is frequently used in the study of evolutionary and sequence homology [51]. The use of *ClustalW* tool enables us to compare and analyze sequence similarity among ESTs simultaneously.

In our case, the multiple sequence alignment is done in two settings. The first involves multiple sequence alignment of ESTs that are from the same gene (intra-gene) in the T-box family. The other setting is to perform multiple sequence alignment on ESTs that are from different genes (inter-gene) in the T-box family. The primary objective is to investigate the degree of similarity for intra-gene and inter-gene ESTs. Based on the multiple sequence alignment results delivered by the *ClustalW* tool, we found out that there is one or more highly similar subsequences appearing in ESTs that belong to the same gene. Besides that, highly similar subsequences can also be identified between ESTs from different genes in the T-box family. However, the key difference between them is the length of highly similar subsequences. The multiple sequence alignment results show that the subsequence length in the first setting is longer than the second setting.

The highlighted key difference justifies the better performance of *wcd* in T-box dataset. This is because *wcd* performs clustering based on the similarity of subsequences between ESTs. It will group ESTs to the same cluster if the length of highly similar subsequences between ESTs is at least the window size (100 bp) of the tool. In fact, we found out that most of the highly similar subsequences between ESTs in the same gene have lengths greater than 100 bp. As a result, *wcd* is able to cluster ESTs in the T-box family according to the genes they belong. On the contrary, *wcd* will not group ESTs from different genes in the T-box family into one cluster, even though they share similar subsequences. The result from multiple sequence alignment between ESTs from different genes in the T-box family has indicated that the lengths of similar subsequences between ESTs are shorter than the window length of *wcd*, and these similarities are not significant to *wcd*. As a result, *wcd* is able to cluster these ESTs correctly.

We further analyze the clustering result of *hbd_EST* in the T-box dataset, it shows that the *hbd_EST* is capable to cluster ESTs to their corresponding genes, but it further partitions the ESTs in the same gene into two or three smaller clusters. It means that the clustering performed by *hbd_EST* is stringent until it can subdivide the members in a gene/cluster into smaller clusters. This could occur when stringent criteria or rules are applied in clustering. Consequently, the clustering accuracy of *hbd_EST* in T-box dataset is lower than *wcd*.

In order to verify the effectiveness of *hbd_EST* on solving the gene family problem in EST clustering, additional statistical tests were conducted. In this case, we performed t-test on the clustering results between *hbd_EST* with *wcd* and *PEACE*. The p-values of the statistical test are displayed in Table 5. The p-value obtained between the *hbd_EST* and *wcd* is 0.0314 (below the alpha of 0.05), and it indicates that both methods have significant difference in terms of clustering accuracy. Another p-value (0.000055) in the table also shows that there is a significant difference between the clustering results of *hbd_EST* and *PEACE*. As such, the statistical test has confirmed that the *hbd_EST* is effective to resolve the stated problem.

This comparative evaluation and statistical test results show that the *hbd_EST* performs quite favorably in the experimental datasets. These results have supported the claim that the *hbd_EST* is effective (in terms of clustering accuracy) for the clustering of ESTs originate from the same gene family. However, it may not perform excellently in all EST datasets. Based on the evaluation results reported in [12], [18], [19], in fact it is very unlikely a clustering method can outperform other methods in all experimental datasets. This is acceptable because there is no unifying theory in clustering [52], [53]. In this case, it means that there is no single distance measure, algorithm or approach for clustering that can be regarded as suitable for most situations [54]. Many techniques or algorithms are designed mainly for solving individual problems.

**Figure 9:**
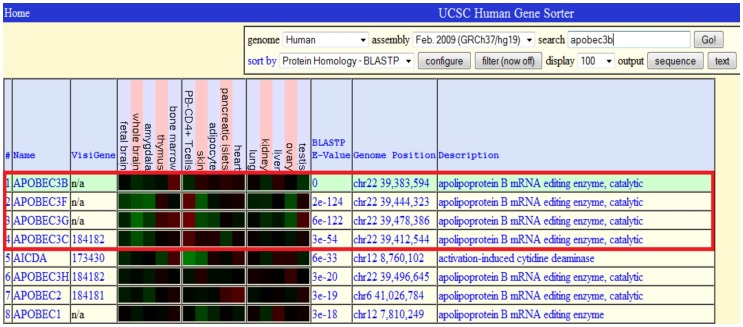
Comparison of protein homology between the APOBEC3B gene and the other genes.

### Evaluation of the Hybrid Distance Measure Using Hierarchical Clustering

We also study the applicability and performance of *hbd_EST* on other clustering techniques. To this end, we select the hierarchical clustering. This is because it is quite a commonly used agglomerative clustering algorithm in EST [17], [18], [47]. The setting of global-local weight in the hybrid distance measure is exactly the same as in previous subsection, they are 0.1 for global and 0.9 for local respectively. Table 6 shows the comparison of clustering accuracies between the DBSCAN and the hierarchical clustering methods.

From the table, nearly equivalent clustering accuracies are observed for the nine datasets, and the only exception is the T-box dataset. The clustering accuracy recorded by hierarchical clustering on T-box dataset is higher than the DBSCAN, where the clustering accuracy is vary by about 0.09. We further conduct the t-test to verify whether or not the two clustering methods have any significant difference in terms of clustering accuracy for the ten datasets. The obtained p-value is 0.37, which is above the alpha of 0.05. This result supports that the two clustering methods have no significant difference in terms of clustering accuracy. Based on the supports from the clustering and t-test results, we can claim that the hybrid distance measure can work quite impressively using the DBSCAN or hierarchical clustering algorithm.

## Discussion

We analyze the cluster membership assignments of the three methods thoroughly, we found out that the lower clustering results obtained by the EST clustering tools are mainly due to the incorrect merging of genes in the datasets. It means that ESTs from two or more genes that are supposed to be clustered separately are now grouped into a single cluster by the clustering tools. For *PEACE* tool, the clustering accuracies for all the datasets are in the range of 0.35–0.70. We investigate its cluster membership assignments properly, and we notice that the incorrect merging incidents occur in every experimental dataset. The occurrences of these incidents have caused the number of clusters generated by the clustering tool are less than expected. This phenomenon (fewer than expected number of clusters) can be observed in Table 4.

The main reason for causing the incorrect merging incidents in the datasets is the high similarity between ESTs from different genes in the family. This is justified because genes are classified into the same family if they share important characteristics or similar functions, and normally they exhibit high degree of sequence homologies [28]. More specifically, they are in fact having similar exon-intron structure and are subsequently differentiated through splicing or evolutionary events [55], [56], [57], [58]. In this case, it means that ESTs from different genes in the same family might share exons among them. As a result, it is difficult to separate these ESTs to the clusters where they belong by using the EST clustering tools that implementing the window-based distance measure. These clustering tools tend to put them to the same cluster even when they share only a single exon region (a subsequence).

For verification, we use the BLAT search for comparing similarities among the ESTs. The BLAT [45] is an alignment search algorithm that is similar to BLAST and it is available in the Genome Browser. The comparisons only focus on ESTs that belong to the incorrect merging incidents. The BLAT search results have confirmed that the incorrect merging incidents are caused likely by the high similarities between ESTs, to an extent where the EST clustering tools are not sensitive enough to separate them. Figure 8 shows an example from the CYP4 dataset. Three ESTs (AI078080, CB163562 and EL735375) from the CYP4A11 gene (position: 47, 394, 848–47, 407, 156, at chromosome 1) are compared against the human genome using the BLAT search.

The search result is displayed in Figure 8A. It shows that the three ESTs obtain the highest matching scores in its own gene, but at the same time they also get the second highest scores with the CYP4A22 gene (position: 47, 603, 107–47, 614, 526, at chromosome 1). Both scores (1^st^ and 2^nd^ highest) only differ slightly, as compared to other scores. This information indicates that both genes are in fact quite similar. Later three ESTs (AA913522, DB187797 and DR004079) from the CYP4A22 gene are also used to conduct the same search, and the result is shown in Figure 8B. We can observe that the result is similar to the previous one, where each EST sets the highest score in its own gene and records the second highest in the CYP4A11 gene.

We further examine the genes using the gene sorter function in the Genome Browser, and they are sorted based on the protein homology. We discover that the genes that tend to be clustered together are relatively similar in terms of protein expression. Figure 9 illustrates the protein homology between the APOBEC genes in the second dataset, and they are measured using the BLASTP E-value (Smaller E-value indicates higher protein similarity between them). The highlighted area (red rectangle) shows that APOBEC3B is quite similar to APOBEC3F, APOBEC3G and APOBEC3C. Therefore, these genes appear as one cluster in the clustering results of both EST clustering tools.

Our proposed hybrid distance measure is able to improve the EST clustering accuracy by separating the genes into different clusters even though they are highly similar. We investigate this matter thoroughly by looking at the proposed hybrid distance measure and the clustering algorithm. We can say that the major contribution is in fact coming from the hybrid distance measure, where it incorporates global and local features into the distance measure. The contribution of the selected clustering algorithm is not as significant as the former, since we have verified that the same distance measure can also produces nearly equivalent clustering results with hierarchical clustering.

The hybrid distance measure is claimed to be quite effective in resolving the gene family problem, and it can be justified with the following explanations. (1) The global feature of EST is measured statistically based on the entire sequence length, therefore it is representing the characteristics of an EST sequence. As a matter of fact, many alignment-free biological sequence comparisons are implemented using the global features [26], [27], [31], [59], [60], [61], [62], thus it is considered influential in sequence comparison. With the incorporation of this global feature into the proposed distance measure, the comparison between two ESTs can be done in a comprehensive manner (global and local scales). As a result, the hybrid distance measure can work satisfactorily on ESTs with high local similarities.

(2) In EST clustering, it only groups ESTs from the same gene and overlap (with specific length) with each other into the same cluster [6], [17]. Hence, the local feature is still the main priority in clustering and the global feature is meant to increase its sensitivity for resolving the gene family problem. In this case, the weight for the local feature is always higher than the global feature. (3) Working on only global or local feature is unable to solve the gene family problem effectively. It has to combine the local feature (to detect similar exon/subsequence between ESTs) and global feature (to differentiate ESTs that share a subsequence but not from the same gene) for accomplishing the clustering task.

In conclusion, we have proposed a hybrid distance measure, *hbd_EST* to improve the clustering accuracy for ESTs that come from the same gene family. This distance measure combines the global and local features extracted from the EST, and the clustering is implemented using the DBSCAN algorithm. The optimized weight for the combined features are obtained from the experimental results, and the best performances are achieved using 0.05–0.15 for the global feature, and 0.85–0.95 for the local feature. The clustering results show that the hybrid distance measure is effective to resolve the problem stated earlier in the paper, where it managed to separate genes from the same family to different clusters. We have demonstrated that this hybrid distance measure is appropriate to be implemented on EST datasets that originating from the same gene family.

Another potential application for *hbd_EST* is for cluster refinement. In this case, a clustering tool can be used to perform coarse clustering for ESTs in a dataset. As a result, ‘super-clusters’ are produced and they can be broken to separate clusters by re-clustering using the *hbd_EST.* Future work includes evaluating the performances (clustering accuracy and speed) of the hybrid distance measure using large EST datasets that contain few millions ESTs. We might also consider implementing it in a parallel environment when it is working with large EST datasets.

## References

[1] He W, Rao Z, Zhou D, Zheng S, Xu W, et al (2012) Analysis of expressed sequence tags and characterization of a novel gene, Slmg7, in the midgut of the common cutworm, Spodoptera litura. PLoS ONE 7(3): e33621. 22470457 10.1371/journal.pone.0033621 PMC3314667

[2] Hazelhurst S, Liptak Z (2011) KABOOM! A new suffix array based algorithm for clustering expression data. Bioinformatics 27(24): 3348–3355. 21984769 10.1093/bioinformatics/btr560

[3] Manickavelu A, Kawaura K, Oishi K, Shin-I T, Kohara Y, et al (2012) Comprehensive functional analyses of expressed sequence tags in common wheat (Triticum aestivum). DNA Research 19(2): 165–177. 22334568 10.1093/dnares/dss001 PMC3325080

[4] Orsini L, Jansen M, Souche E, Geldof S, Meester LD (2011) Single nucleotide polymorphism discovery from expressed sequence tags in the water flea Daphnia magna. BMC Genomics 12: 309. 21668940 10.1186/1471-2164-12-309 PMC3146954

[5] Shen Y, Burger G (2010) TestLoc: protein subcellular localization prediction from EST data. BMC Bioinformatics 11: 563. 21078192 10.1186/1471-2105-11-563 PMC3000424

[6] Nagaraj SH, Gasser RB, Ranganathan S (2006) A hitchhiker’s guide to expressed sequence tag (EST) analysis. Brief in Bioinformatics 8: 6–21. 16772268 10.1093/bib/bbl015

[7] Boguski MS, Schuler GD (1995) ESTablishing a human transcript map. Nature Genet 10: 369–371. 7670480 10.1038/ng0895-369

[8] Quackenbush J, Cho J, Lee D, Liang F, Holt I, et al (2001) The TIGR gene indices: analysis of gene transcript sequences in highly sampled eukaryotic species. Nucleic Acids Res 29: 159–164. 11125077 10.1093/nar/29.1.159 PMC29813

[9] Miller RT, Christoffels AG, Gopalakrishnan C, Burke J, Ptitsyn AA, et al (1999) A comprehensive approach to clustering of expressed human gene sequence: the sequence tag alignment and consensus knowledge base. Genome Res 9: 1143–1155. 10568754 10.1101/gr.9.11.1143 PMC310831

[10] Kent WJ, Sugnet CW, Furey TS, Roskin KM, Pringle TH, et al (2002) The human genome browser at UCSC. Genome Res 12: 996–1006. 12045153 10.1101/gr.229102 PMC186604

[11] Altschul SF, Gish W, Miller W, Myers EW, Lipman DJ (1990) Basic local alignment search tool. J Mol Biol 215: 403–410. 2231712 10.1016/S0022-2836(05)80360-2

[12] Kalyanaraman A, Aluru S, Kothari S, Brendel V (2003) Efficient clustering of large EST data sets on parallel computers. Nucleic Acids Res 31: 2963–2974. 12771222 10.1093/nar/gkg379 PMC156714

[13] Wu TD, Watanabe CK (2005) GMAP: a genomic mapping and alignment program for mRNA and EST sequences. Bioinformatics 21: 1859–1875. 15728110 10.1093/bioinformatics/bti310

[14] Wu X, Lee W, Gupta D, Tseng C (2005) ESTMapper: efficiently clustering EST sequences using genome maps. Proc of the 19^th^ IEEE Int. Parallel and Distributed Processing Symposium: 196a.

[15] Kim N, Shin S, Lee S (2005) ECgene: genome-based EST clustering and gene modeling for alternative splicing. Genome Res 15: 566–576. 15805497 10.1101/gr.3030405 PMC1074371

[16] Picardi E, Mignone F, Pesole G (2009) EasyCluster: a fast and efficient gene-oriented clustering tool for large-scale transcriptome data. BMC Bioinformatics 10: S10. 10.1186/1471-2105-10-S6-S10 PMC2697633 19534735

[17] Burke J, Davidson D, Hide W (1999) d2_cluster: a validated method for clustering EST and full-length cDNA sequences. Genome Res 9: 1135–1142. 10568753 10.1101/gr.9.11.1135 PMC310833

[18] Hazelhurst S, Hide W, Liptak Z, Nogueira R, Starfield R (2008) An overview of the wcd EST clustering tool. Bioinformatics 24: 1542–1546. 18480101 10.1093/bioinformatics/btn203 PMC2718666

[19] Rao DM, Moler JC, Ozden M, Zhang Y, Liang C, et al (2010) PEACE: parallel environment for assembly and clustering of gene expression. Nucleic Acids Res 38: 737–742. 10.1093/nar/gkq470 PMC2896108 20522511

[20] Christoffels A, Gelder A, Greyling G, Miller R, Hide T, et al (2001) STACK: sequence tag alignment and consensus knowledgebase. Nucleic Acids Res 29: 234–238. 11125101 10.1093/nar/29.1.234 PMC29830

[21] Pevzner PA (1992) Statistical distance between texts and filtration methods in sequence comparison. Comp Appl Biosci 8: 121–127. 1591607 10.1093/bioinformatics/8.2.121

[22] Blasdell BE (1986) A measure of similarity of sets of sequences not requiring sequence alignment. Proc Natl Acad Sci 83: 5155–5159. 3460087 10.1073/pnas.83.14.5155 PMC323909

[23] Mantaci S, Restivo A, Rosone G, Sciortino M (2008) A new combinatorial approach to sequence comparison. Theory of Computing Systems 42: 411–429.

[24] Otu HH, Sayood K (2003) A new sequence distance measure for phylogenetic tree construction. Bioinformatics 19: 2122–2130. 14594718 10.1093/bioinformatics/btg295

[25] Dai Q, Wang T (2008) Comparison study on k-word statistical measures for protein: From sequence to sequence space. BMC Bioinformatics 9: 394. 18811946 10.1186/1471-2105-9-394 PMC2571980

[26] Almeida JS, Vinga S (2002) Universal sequence map (USM) of arbitrary discrete sequences. BMC Bioinformatics 3: 6. 11895567 10.1186/1471-2105-3-6 PMC90187

[27] Wu TJ, Hsieh YC, Li LA (2001) Statistical measures of DNA sequence dissimilarity under Markov chain models of base composition. Biometrics 57: 441–443. 11414568 10.1111/j.0006-341x.2001.00441.x

[28] Demuth JP, Bie TD, Stajich JE, Cristianini N, Hahn MW (2006) The evolution of mammalian gene families. PLoS ONE 1: e85. 17183716 10.1371/journal.pone.0000085 PMC1762380

[29] Ruan J, Li H, Chen Z, Coghlan A, Coin LJ, et al (2008) TreeFam: 2008 update. Nucleic Acids Res 36: D735–D740. 18056084 10.1093/nar/gkm1005 PMC2238856

[30] Pham TD, Zuegg J (2004) A probabilistic measure for alignment-free sequence comparison. Bioinformatics 20: 3455–3461. 15271780 10.1093/bioinformatics/bth426

[31] Wu TJ, Burke JP, Davidson DB (1997) A measure of DNA sequence dissimilarity based on Mahalanobis distance between frequencies of words. Biometrics 53: 1431–1439. 9423258

[32] Han J, Kamber M (2006) Data preprocessing, in: Data mining: concepts and techniques. San Francisco: Morgan Kaufmann. 47–97.

[33] Ankerst M, Breunig MM, Kriegel H, Sander J (1999) OPTICS: ordering points to identify the clustering structure. Proc ACM SIGMOD ’99 Int Conf on Management of Data: 49–60.

[34] Agrawal R, Gehrke J, Gunopulos D, Raghavan P (1998) Automatic subspace clustering of high dimensional data for data mining applications. Proc ACM SIGMOD ’98 Int Conf on Management of Data: 94–105.

[35] Ester M, Kriegel H, Sander J, Xu X (1996) A density-based algorithm for discovering clusters in large spatial databases with noise. Proc of 2^nd^ Int Conf on Knowledge Discovery and data mining: 226–231.

[36] Ruiz C, Spiliopoulou M, Menasalvas E (2010) Density-based semi-supervised clustering. Data mining and knowledge discovery 21: 345–370.

[37] Nasibov E, Ulutagay G (2009) Robustness of density-based clustering methods with various neighborhood relations. Fuzzy Sets and Systems 160: 3601–3615.

[38] Gorawski M, Malczok R (2006) AEC algorithm: a heuristic approach to calculating density-based clustering Eps parameter. Advances in Information Systems LNCS 4243: 90–99.

[39] Chen Y, Relly KD, Sprague AP, Guan Z (2006) SEQOPTICS: a protein sequence clustering system. BMC Bioinformatics 7: S10. 10.1186/1471-2105-7-S4-S10 PMC1780130 17217502

[40] Halkidi M, Batistakis Y, Vazirgiannis M (2002) Cluster validity methods: Part I. SIGMOD Rec. 31: 40–45.

[41] Malde K, Coward E, Jonassen I (2003) Fast sequence clustering using a suffix array algorithm. Bioinformatics 19: 1221–1226. 12835265 10.1093/bioinformatics/btg138

[42] Seal RL, Gordon SM, Lush MJ, Wright MV, Bruford EA (2011) Genenames.org: the HGNC resources in 2011. Nucleic Acids Res 39: 514–519. 10.1093/nar/gkq892 PMC3013772 20929869

[43] Karolchik D, Hinrichs AS, Furey TS, Roskin KM, Sugnet CW, et al (2004) The UCSC table browser data retrieval tool. Nucleic Acids Res 32: 493–496. 10.1093/nar/gkh103 PMC308837 14681465

[44] Pruitt KD, Tatusova T, Klimke W, Maglott DR (2009) NCBI reference sequences: current status, policy, and new initiatives. Nucleic Acids Res 37: 32–36. 10.1093/nar/gkn721 PMC2686572 18927115

[45] Kent WJ (2002) BLAT–the Blast-like alignment tool. Genome Res 12: 656–664. 11932250 10.1101/gr.229202 PMC187518

[46] Hazelhurst S (2008) Algorithms for clustering expressed sequence tags: the wcd tool. South African Computer Journal 40: 51–62.

[47] Ptitsyn A, Hide W (2005) CLU: a new algorithm for EST clustering. BMC Bioinformatics 6(2): S3. 10.1186/1471-2105-6-S2-S3 PMC1637039 16026600

[48] Mellas DC, Schouls L, Francois P, Herzig S, Verbrugh HA, et al (2009) High-throughput typing of Staphylococcus aureus by amplified fragment length polymorphism (AFLP) or multi-locus variable number of tandem repeat analysis (MLVA) reveals consistent strain relatedness. Eur J Clin Microbiol Infect Dis 28: 39–45. 18663501 10.1007/s10096-008-0585-4

[49] Curtis AJ (2008) Three-dimensional visualization of cultural clusters in the 1878 yellow fever epidemic of New Orleans. International Journal of Health Geographics 7: 47. 18721469 10.1186/1476-072X-7-47 PMC2538514

[50] Larkin MA, Blackshields G, Brown NP, Chenna R, McGettigan PA, et al (2007) ClustalW and ClustalX version 2. Bioinformatics 23: 2947–2948. 17846036 10.1093/bioinformatics/btm404

[51] Edgar RC, Batzoglou S (2006) Multiple sequence alignment. Current Opinion in Structural Biology 16: 368–373. 16679011 10.1016/j.sbi.2006.04.004

[52] Yang Q, Wu X (2006) 10 challenging problems in data mining research. International Journal of Information Technology & Decision Making 5(4): 597–604.

[53] Gokcay E, Principe JC (2002) Information Theoretic Clustering. IEEE Transactions on Pattern Analysis and Machine Intelligence 24: 158–171.

[54] Milligan GW (1996) Clustering validation: Results and implications for applied analysis. In: Arabie P, Hubert LJ, De Soete G, editors. Clustering and Classification. River Edge NJ: World Scientific Publ. 341–375.

[55] Peremyslov VV, Mockler TC, Filichkin SA, Fox SE, Jaiswal P, et al (2011) Expression, splicing and evolution of the myosin gene family in plants. Plant Physiology 155: 1191–1204. 21233331 10.1104/pp.110.170720 PMC3046578

[56] Itoh N, Ornitz DM (2008) Functional evolutionary history of the mouse *Fgf* gene family. Development Dynamics 237(1): 18–27. 10.1002/dvdy.21388 18058912

[57] Rogozin IB, Sverdlov AV, Babenko VN, Koonin EV (2005) Analysis of evolution of exon-intron structure of eukaryotic genes. Briefings in Bioinformatics 6: 118–134. 15975222 10.1093/bib/6.2.118

[58] Sanchez D, Ganfornina MD, Gutierrez G, Marin A (2003) Exon-intron structure and evolution of the Lipocalin gene family. Molecular Biology and Evolution 20(5): 775–783. 12679526 10.1093/molbev/msg079

[59] Afreixo V, Bastos CA, Pinho AJ, Garcia SP, Ferreira PJ (2009) Genome analysis with inter-nucleotide distances. Bioinformatics 25(23): 3064–3070. 19759198 10.1093/bioinformatics/btp546 PMC2778338

[60] Li M, Chen X, Li X, Ma B, Vitanyi P (2004) The similarity metric. IEEE Transactions on Information Theory 50(12): 3250–3264.

[61] Stuart GW, Moffett K, Baker S (2002) Integrated gene and species phylogenies from unaligned whole genome protein sequences. Bioinformatics 18: 100–108. 11836217 10.1093/bioinformatics/18.1.100

[62] Li M, Badger JH, Chen X, Kwong S, Kearney P, et al (2001) An information-based sequence distance and its application to whole mitochondrial genome phylogeny. Bioinformatics 17: 149–154. 11238070 10.1093/bioinformatics/17.2.149

